# Understanding the participation of breast screening among women born in predominantly Muslim countries living in Victoria, Australia from record-linkage data

**DOI:** 10.1371/journal.pone.0237341

**Published:** 2020-08-07

**Authors:** Tahira Yeasmeen, Margaret Kelaher, Julia M. L. Brotherton, Michael J. Malloy

**Affiliations:** 1 Centre for Health Policy, Melbourne School of Population and Global Health, The University of Melbourne, Parkville, Victoria, Australia; 2 VCS Population Health, VCS Foundation, East Melbourne, Victoria, Australia; 3 VICNISS, Melbourne Health, Melbourne, Victoria, Australia; Ravensburg-Weingarten University of Applied Sciences, GERMANY

## Abstract

**Background:**

Early detection of breast cancer can improve survival rates and decrease mortality rates. This study investigates whether there are significant differences in participation in breast screening among women born in Muslim countries compared to women born in Non-Muslim countries and Australia.

**Methods:**

Screening data from January 1^st^, 2000 to December 31^st^, 2013 from the Breast Screen Victoria Registry (BSV) was linked with hospital records from the Victorian Admitted Episodes Dataset (VAED). Countries having more than 50% of their population as Muslim were categorised as Muslim countries. Age adjusted rates were calculated for women born in Muslim and Non-Muslim countries and compared with the Australian age adjusted rates. Logistic regression assessed the association between screening status and other factors which include country of birth, marital status, age and socio-economic status.

**Results:**

Women born in Muslim countries (Odds Ratio (OR) = 0.70, 95%CI = 0.68–0.72) and in other Non-Muslim countries (OR = 0.87, 95%CI = 0.86–0.88) had lower odds of participation in breast screening than Australian born women. Women aged 60–64 years (OR = 1.42, 95%CI = 1.40–1.44) had higher odds of participation in the BreastScreen program than 50–54 age group.

**Conclusion:**

This study provides valuable insights to understanding breast screening participation among women born in Muslim countries residing in Victoria. This population level study contributes to the broader knowledge of screening participation of women born in Muslim countries, an understudied population group in Australia and across the world. This study has implications for breast screening programs as it highlights the need for culturally sensitive approaches to support breast screening participation among women born in Muslim countries.

## Introduction

Breast cancer is one of the most common cancers and major public health concerns worldwide. In 2018, an estimated 2.09 million new breast cancer cases (11.6% of all cancers) were diagnosed worldwide [1]. In general, high income countries such as the U.S., England and Australia have higher incidence rates of breast cancer than lower income countries [1]. However, breast cancer is the most common cause of cancer mortality among women in low and middle income countries and is the second most common cause of cancer mortality in high income countries after lung cancer [2]. Early detection of cancer can improve survival rates and decrease mortality rates. For instance, screening mammography reduces the risk of death from breast cancer by 40% [3]. To reduce morbidity and mortality rates from breast cancer, BreastScreen Australia operates through organised screening by enabling early intervention and recommends a mammogram every two years for all women 50 to 74 years of age [4]. Thus, screening is a significant public health measure to lower the mortality and morbidity of breast cancer among women in many countries through organised screening programs [5].

Previous studies in various western countries found that cancer screening participation is strongly associated with racial and/or ethnic backgrounds. For instance, an Australian study found that women from East Asia and the Middle East/North Africa were less likely to participate in breast screening than Australian-born women [6]. Renshaw et al. [7] found that in the UK, White women were more likely to obtain a mammogram than Black and Asian women. In the U.S., Hispanic women participate in breast screening services less than African-American and White women [8]. A Canadian study found that breast screening uptake is low among immigrant and minority women compared to Canadian born women [9]. In the US, recent studies have found that there were significant differences in the mortality rates and the diagnosis of breast cancer among Muslim women. Positive religious coping (e.g., use of religious support such as prayer to overcome stresses) and lower self-efficacy [10] were associated with lower rates of having a mammogram among American Muslim women.

To investigate the extent to which Muslim women participate in cancer screening in Australia, instead of correlating religion with cancer screening, studies have shown how screening practices differ based on country of birth. Using the 1995 Australian National Health Survey data, Siahpush and Singh [11] found that women born in Southern Europe, the Middle East, and Asia were less likely to participate in mammographic screening compared to those born in Australia/New Zealand. Few research [10,12] have studied how Muslim women participate in breast screening. These studies are limited to small scale survey data on screening participation.

In this paper, we present an analysis of record linkage data from Victoria, Australia to understand the breast screening participation status of women born in predominantly Muslim countries (hereafter women born in Muslim countries). Muslim population in Australia is growing with diverse socio-cultural backgrounds. Islam is one of the rapidly growing religions in Australia. Around 62 percent of the Muslims who reside in Australia were born overseas leading to significant ethnic and racial diversity among Muslim immigrant communities [13]. Previous studies have found that health behaviour of the Muslim population is influenced by values formed by their religious affiliation [14]. In our prior study [15], using cervical screening registry data matched with hospital admission records, we also observed that women born in Muslim countries participate less in cervical screening than women born in Australia and other Non-Muslim countries. In that study, we used Victorian Admitted Episodes Dataset (VAED) linked with Victorian Cervical Cytology Registry (VCCR) data and estimated the cervical screening rates for women in Victoria. In this study, the same base cohort (VAED) is used; however, we linked Victorian breast screening registry data with this base cohort. Here we investigate if women born in Muslim countries participate less in breast screening than the women born in other countries (e.g., Australia and Non-Muslim countries). This study contributes towards understanding breast screening participation of women born in Muslim countries across various racial and ethnic groups present in the community. While previous studies were based on survey data with limited sample size, this study considers Victorian breast screening registry data which includes all women participated in breast screening program in Victoria, Australia. This study provides broader insights on how women born in Muslim countries, a population group not widely studied in the literature, participate in breast screening.

## Materials and methods

### Sample

To estimate breast screening participation among women by country of birth, screening data was obtained from the Breast Screen Victoria Registry (BSV) and linked with hospital records from the Victorian Admitted Episodes Dataset (VAED), which routinely collects country of birth information. This enabled us primarily to determine whether or not women, who were Victorian residents admitted to hospital on one or more occasions over a 14-year period, participated in the BSV program according to their hospital collected country of birth information. The VAED is our base cohort.

The Victorian Admitted Episodes Dataset (VAED) is a comprehensive dataset which consists of the causes, effects and nature of illness, and the use of health services in Victoria. The VAED facilitates to plan health services, formulate health care policies, conduct epidemiological research, and provide public hospital funds. The dataset is created based on the information provided by the hospitals. All the hospitals in Victoria both public and private, publish a minimum dataset for each admitted patient episode. This also include the rehabilitation centres, extended care facilities and day procedure centres (Department of Health and Human Services, 2020).

The objective of this study was to determine the breast screening participation status of women born in Muslim countries. The VAED dataset is a complete and comprehensive, systematically collected state-wide data set of all admissions in Victoria allowing us to assemble a very large cohort of women from the Victorian community with country of birth information. Linking the VAED dataset with the Breast Screen Victoria Registry allowed us to determine the women who were admitted to the hospital and participated in breast screen program and obtain their country of birth information. Thus, we chose the VAED dataset as our base cohort to acquire the country of birth information.

The VAED and BSV data sets included records spanning the time period January 1^st^, 2000 to December 31^st^, 2013 for women aged 45 years to 84 years. Following data custodian and ethical approval (Ethics application approved by Department of Health Human Research Ethics Committee (HRECs), Ethic Approval Reference- ADF/11/4510), the Victorian Data Linkage (VDL) unit used a standard probabilistic data linkage protocol, with separation of identifying linkage variables from analytic variables in each data set designed to preserve privacy. Variables used for linkage were date of birth, sex, postcode, and Medicare number (Australia’s universal health insurance identifier); name and address were not available in the VAED dataset. However, as BSV doesn’t collect Medicare number and to increase matching accuracy, the BSV data was first linked to the Victorian Cervical Cytology Registry (VCCR) database as it routinely collects this information.

Following data linkage, VDL advised us which records from each data set belonged together via anonymised linkage keys. Records from VAED and BSV with linkage keys that merged successfully were women who had apparently screened. We use the term ‘Apparent Screening Rates (ASR)’ to reflect that the data linkage process will underestimate actual screening rates due to the limitations of the available identifying variables.

Analytic variables from BSV include details of each screening episode, statistical local area (SLA) information, country of birth, and age at time of each test. Variables from VAED include country of birth, marital status, preferred language, age at each admission, principal diagnosis (by ICD10AM code), insurance, statistical local area (SLA) information and hospital status. For the women who screened, we used the ‘Country of Birth’ at the time of screening (obtained from the BSV data) and for those who didn’t screen, we used the country of birth from their most recent hospital admission.

We classified women according to their country of birth and referred as women born in Muslim countries, women born in Non-Muslim countries and women born in Australia. Muslim countries were defined by utilizing the percentage of Muslim population derived from The Pew Research Center [16]. Among the total population of the respective countries, if 50% or more of their population are Muslim then those countries were categorised as Muslim countries. We have added the names of the countries labelled as Muslim in Appendix A.

The age of each woman in the linked data set was calculated at the mid-point (31st December 2012) of our analysis period (2012–2013). This time period was selected as the most recent 2-year screening period in the linked data set and therefore most likely to reflect current screening rates to inform policy makers. If a woman had multiple screens within this time, her first screening date was used. Women who had an age difference of more than one year between VAED and BSV data at the same time point were excluded from the cohort as they were possibly not the same individual. Women who were discharged from the screening program (due to a cancer diagnosis or being unsuitable for general risk population screening) or died prior to the time period of interest were also excluded.

We derived the socio-economic status of a woman by assigning the area level Socio-Economic Indexes for Areas (SEIFA) for her SLA, obtained from the Australian Bureau of Statistics (ABS). In particular, to derive the socio-economic status variable, we used the Index of Relative Socio-Economic Advantage and Disadvantage (IRSAD Deciles) [17] and classified it into five categories: Low, Low-Medium, Medium, High Medium, and High. Marital status was divided into seven categories: Never Married, Widowed, Divorced, Separated, Married, De Facto, and Not Stated/Inadequate.

Following completion of the linkage study and analysis, we successfully obtained unpublished deidentified data from the BSV Register (Ethics application approved by Melbourne School of Population and Global Health (MSPGH) Human Ethics Advisory Group (HEAG), Ethics ID: 1954010.1) including self-reported country of birth for women age 50–74 attending BSV for screening in 2016–2017. We used denominator estimates for the number of women in the relevant age groups in Victoria by country of birth from the 2016 Australian census [18]. Because of concerns about the validity of estimates for countries with small numbers, we grouped them to minor and major countries as per the ABS standard geographical classification. BSV does not routinely publish participation estimates by country of birth due to the challenges in obtaining accurate population denominator data by country of birth over time and for small areas. We then used these data to directly estimate breast screening participation of women born in Muslim and non-Muslim countries to validate and compare our results from the linkage data.

### Ethics approval

Ethics application approved for this project by Department of Health Human Research Ethics Committee (HRECs), Ethics ID- ADF/11/4510 and Melbourne School of Population and Global Health (MSPGH) Human Ethics Advisory Group (HEAG), Ethics ID: 1954010.1.

### Statistical methods

We report the descriptive statistics estimating the percentage of women in the hospital cohort for different age ranges, marital status, socio-economic status, and screening participation. We calculated ASR for breast cancer for the two-year time period of 2012–2013. That is, the percentage of women who have participated in BSV’s screening program as identified through data linkage among the women eligible for screening within the VAED cohort. We estimated logistic regression models to understand the association between screening status and other factors including country of birth, marital status, age, and socio-economic status. We calculated age adjusted rates for women born in Muslim and Non-Muslim countries and compared with the Australian adjusted rates. For every variable of the logistic regression model, there were likelihood ratio tests; which were conducted to compare the goodness of fit of two models. Independent variables used in the multiple logistic regression model included country of birth, marital status, age and socio-economic status and the outcome variable was whether a woman has participated in mammogram screening or not. We used those variables in the model which have high interest and relevance to the study. We did not include other available variables with low completion rates or those not directly relevant to our study. In this paper the statistical analyses were performed using Stata/SE version 12.1.

## Results

The total number of women of the VAED cohort are presented in Table 1 by their age, marital and socio-economic categories and grouped by their country of birth. In Table 1 we see that, 610,114 number of women were born in Australia; 276,161 number of women were born in other Non-Muslim countries; and 32,576 number of women were born in Muslim countries. The age distributions across these three groups were similar except that there were more women who were born in Muslim countries (22%) in age group between 50–54 years than in Australia (19.8%) and in Non-Muslim countries (15.6%) of the same age group. The highest percentage of married women (69.4%) belong to women born in Muslim countries. In terms of socio-economic status, across all the groups of women, most women resided in areas with a high-medium category socio-economic status (27.7% of women were born in Muslim countries, 28.7% of women were born in Australia, and 30.9% of women were born in Non-Muslim countries).

**Table 1:** Descriptive statistics of women of Victoria who were in the hospital admitted at least once during the period 2000–2013 by their country of birth (women who were born in Australia, Muslim and Non–Muslim countries) (Source: Victorian Admitted Episodes Database).

	Australia		Muslim		Non-Muslim	
	N	%	N	%	N	%
**Age**						
45–49	52,374	8.6	3,339	10.2	18,322	6.6
50–54	120,852	19.8	7,167	22.0	43,186	15.6
55–59	104,906	17.2	6,014	18.5	41,060	14.9
60–64	92,672	15.2	5,292	16.2	42,974	15.6
65–69	79,668	13.1	3,915	12.0	41,447	15.0
70–74	59,585	9.8	2,873	8.8	33,914	12.3
75–79	51,089	8.4	2,295	7.0	31,180	11.3
80–84	48,968	8.0	1,681	5.2	24,078	8.7
**Total**	**610,114**	**100.0**	**32,576**	**100.0**	**276,161**	**100.0**
**Marital status**						
Never Married	64,095	10.5	2,209	6.8	17,647	6.4
Widowed	60,060	9.8	4,221	13.0	35,123	12.7
Divorced	50,087	8.2	1,860	5.7	19,359	7.0
Separated	17,015	2.8	765	2.3	6,689	2.4
Married	379,340	62.2	22,623	69.4	186,849	67.7
De Facto	28,884	4.7	565	1.7	7,248	2.6
Not stated/inadequate	10,633	1.7	333	1.0	3,246	1.2
**Total**	**610,114**	**100.0**	**32,576**	**100.0**	**276,161**	**100.0**
**Socioeconomic status**							
Low	64,251	10.5	6,361	19.5	38,504	13.9
Low-Medium	130,806	21.4	5,668	17.4	53,219	19.3
Medium	118,252	19.4	4,195	12.9	39,318	14.2
High-Medium	175,278	28.7	9,023	27.7	85,220	30.9
High	121,527	19.9	7,329	22.5	59,900	21.7
**Total**	**610,114**	**100.0**	**32,576**	**100.0**	**276,161**	**100.0**

Breast cancer ASR for the two-year time period of 2012–2013 included a total of 938,560 women. Table 2 reports the ASR of women who were born in Muslim countries, Australia and other Non-Muslim countries. Women born in Muslim countries had lower ASR (24.8%) in comparison to the women who were born in Australia and other Non-Muslim countries (29.7% and 26.8% respectively). The highest ASR belong to the women who were in the age group of 60–64 years and were born in Muslim (36.7%) and Non–Muslim countries (41.6%) but amongst women born in Australia, women who were in age group of 65–69 years reported to have the highest ASR (43.4%). The trend of Muslim women having lower ASR was consistent across all age groups. For example, amongst women aged 50–54 years, the ASR was lower among women who were born in Muslim countries (29.9%) than for those born in Australian (34.4%) and Non–Muslim countries (33.0%).

**Table 2:** Apparent screening rates (ASR) of hospitalized women by their country of birth (women who were born in Australia, Muslim and Non–Muslim countries) in 2012–2013.

Australia				Muslim				Non- Muslim			
Age	Screened	Total (Pop. dist.^a^)	ASR	Age	Screened	Total (Pop. dist.^a^)	ASR	Age	Screened	Total (Pop. dist.^a^)	ASR
45–49	3,503	52,374 (8.6%)	6.7%	45–49	187	3,339 (10.2%)	5.6%	45–49	1,200	18,322 (6.6%)	6.5%
50–54	41,624	120,852 (19.8%)	34.4%	50–54	2,144	7,167 (22.0%)	29.9%	50–54	14,243	43,186 (15.6%)	33.0%
55–59	42,246	104,906 (17.2%)	40.3%	55–59	2,079	6,014 (18.5%)	34.6%	55–59	15,827	41,060 (14.9%)	38.5%
60–64	39,893	92,672 (15.2%)	43.0%	60–64	1,944	5,292 (16.2%)	36.7%	60–64	17,861	42,974 (15.6%)	41.6%
65–69	34,594	79,668 (13.1%)	43.4%	65–69	1,313	3,915 (12.0%)	33.5%	65–69	16,991	41,447 (15.0%)	41.0%
70–74	13,743	59,585 (9.8%)	23.1%	70–74	328	2,873 (8.8%)	11.4%	70–74	5,824	33,914 (12.3%)	17.2%
75–79	4,866	51,089 (8.4%)	9.5%	75–79	69	2,295 (7.0%)	3.0%	75–79	1,756	31,180 (11.3%)	5.6%
80–84	941	48,968 (8.0%)	1.9%	80–84	9	1,681 (5.2%)	0.5%	80–84	246	24,078 (8.7%)	1.0%
**Total**	**181,410**	**610,114 (100.0%)**	**29.7%**	**Total**	**8,073**	**32,576 (100.0%)**	**24.8%**	**Total**	**73,948**	**276,161 (100.0%)**	**26.8%**

^a^ Pop dist.- Population Distribution

**Fig 1:**
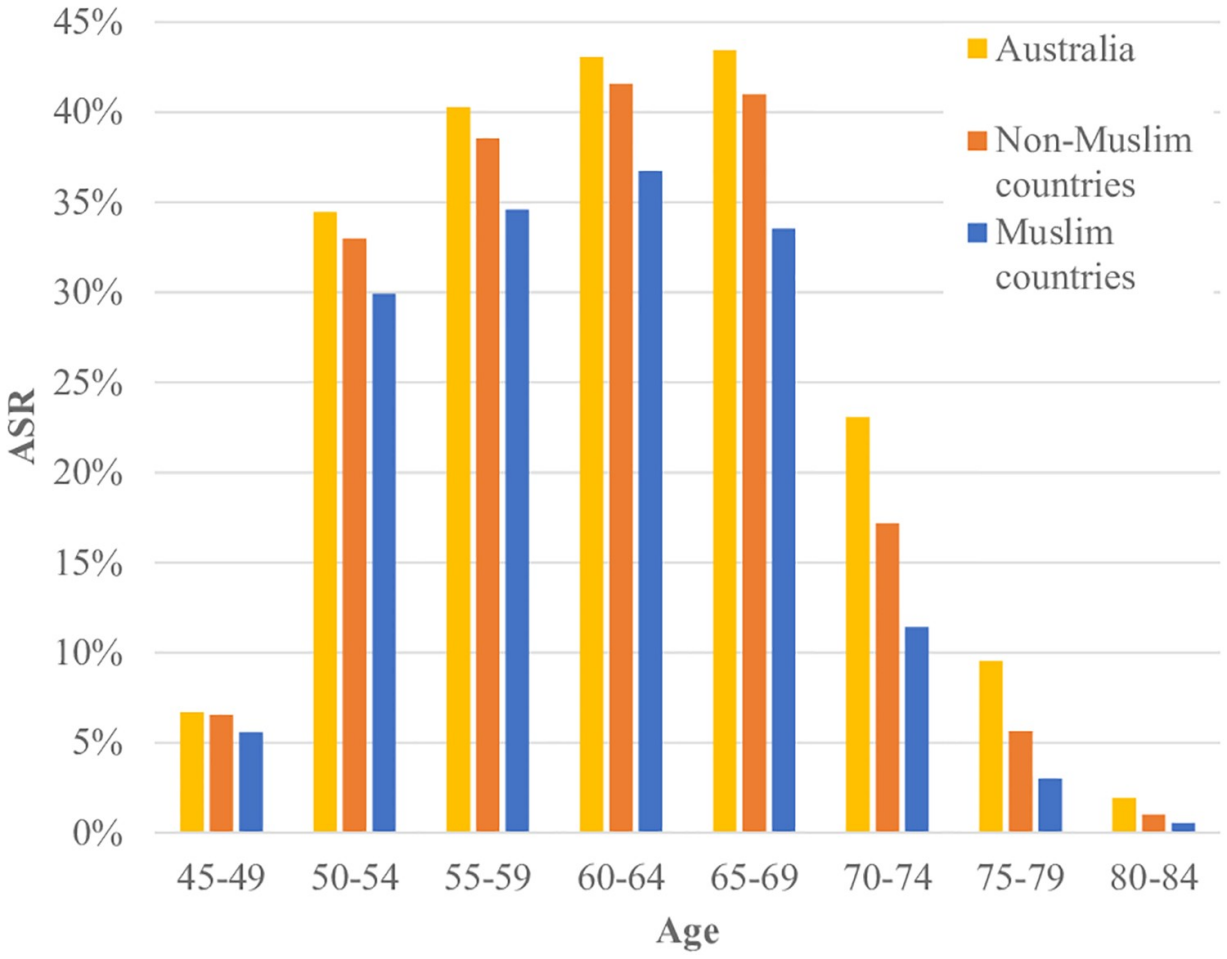
ASR of women who were born in Australia, Muslim and Non–Muslim countries in 2012–2013 by age.

**Fig 2:**
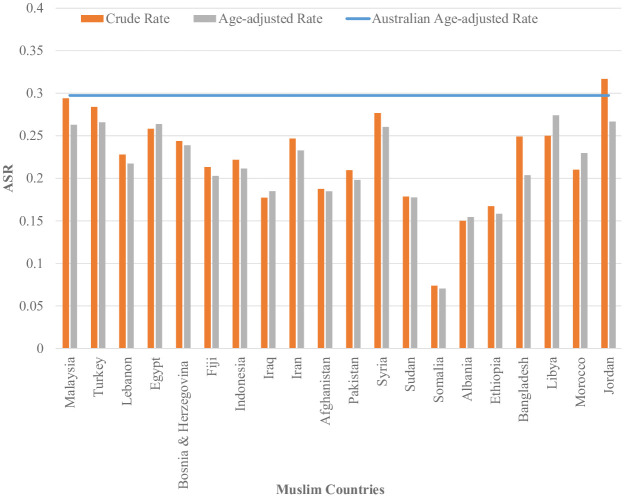
ASR for women who were born in Muslim countries in 2012–2013 adjusted for age (Countries have been arranged by number of women in descending order).

**Fig 3:**
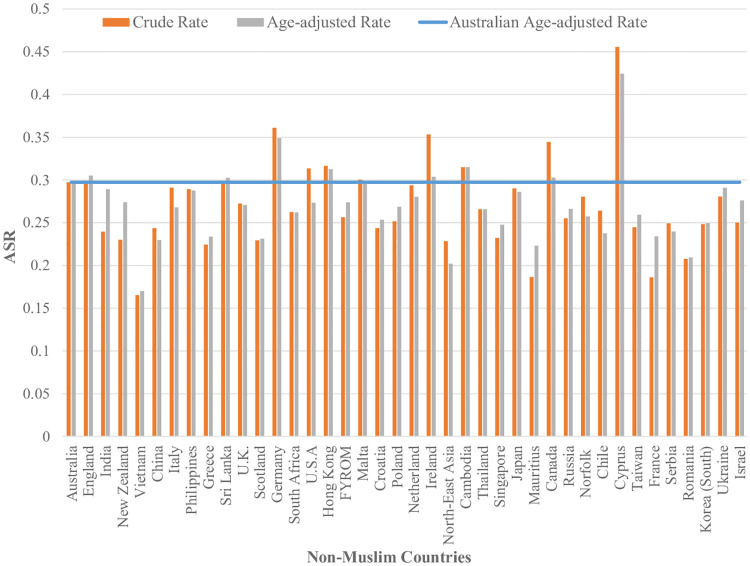
ASR for women who were born in Australia and other Non-Muslim countries in2012-2013 adjusted for age (Countries have been arranged by number of women in descending order).

Fig 1 shows that across all age categories, Australian born women had higher ASR than women who were born in Non-Muslim and Muslim countries. Women who were born in Muslim countries reported to have the lowest ASR across all age categories. We also found that women who were born in all Muslim countries reported to have lower ASR in comparison to the women born in Australia and other Non-Muslim countries. Fig 2 shows that, for twenty Muslim counties, the age-adjusted screening rates for women born in those countries are lower than the Australian age-adjusted rate. In Fig 3 we can see that a few countries have ASR above the Australian adjusted rate (e.g.; Germany, Hong Kong, Ireland, Cambodia, Canada and Cyprus) and most of them have rates closer to the Australian adjusted rate (e.g.; England, Italy, Philippines, Sri Lanka, Malta, Netherland and Japan). India, New Zealand, Vietnam, China, Greece, the U.K., Scotland, South Africa, the USA, Croatia, Poland, Thailand, Singapore, Mauritius, Russia, Chile, Taiwan, France, Serbia, Romania, South Korea and Israel are some of the Non-Muslim countries who have their ASR lower than the Australian adjusted rate.

The multivariate logistic regression model results are presented in Table 3, where the association between screening status and various factors are being considered. The likelihood of being screened are higher among the women who are in the age groups of 60–64 and 65–69 years compared to the women who are in reference age group of 50–54 years. In reference to the high-medium category of socio-economic status, the medium category women have the highest odds ratio to participate in screening. On the other hand, we found that the odds ratios are lower among women who are never married, are divorced, separated or widowed compared to the women who are married. The odds of screening for women who were born in Muslim and other Non-Muslim countries are lower than for women born in Australia. Women who were born in Muslim countries reported to have the lowest likelihood of participating in screening.

**Table 3:** Association of breast screening participation status with factors such as country of birth, marital status, age, and socio-economic status based on a multi-variate logistic regression model (N = 938,560).

Variables	Odds Ratio	95% CI^a^	P-value|
**Country of birth**			
Australian	Reference		
Muslim	0.70	0.68–0.72	<0.001
Non-Muslim	0.87	0.86–0.88	<0.001
Not classified	0.46	0.45–0.48	<0.001
**Marital status**			
Married	Reference		
Never married	0.63	0.62–0.64	<0.001
Widowed	0.82	0.80–0.83	<0.001
Divorced	0.71	0.70–0.73	<0.001
Separated	0.56	0.54–0.58	<0.001
De facto	0.61	0.59–0.62	<0.001
Not stated/inadequately described	1.02	0.98–1.05	0.412
**Age**			
50–54	Reference		
45–49	0.14	0.13–0.14	<0.001
55–59	1.27	1.26–1.29	<0.001
60–64	1.42	1.40–1.44	<0.001
65–69	1.41	1.39–1.43	<0.001
70–74	0.50	0.49–0.51	<0.001
75–79	0.17	0.16–0.17	<0.001
80–84	0.03	0.03–0.03	<0.001
**Socio-economic status**			
High-Medium	Reference		
Low	1.05	1.03–1.06	<0.001
Low-Medium	1.07	1.05–1.08	<0.001
Medium	1.18	1.17–1.20	<0.001
High	0.86	0.84–0.87	<0.001
**Constant**	0.60	0.59–0.61	<0.001

^a^ 95%CI- 95% Confidence Interval

**Fig 4:**
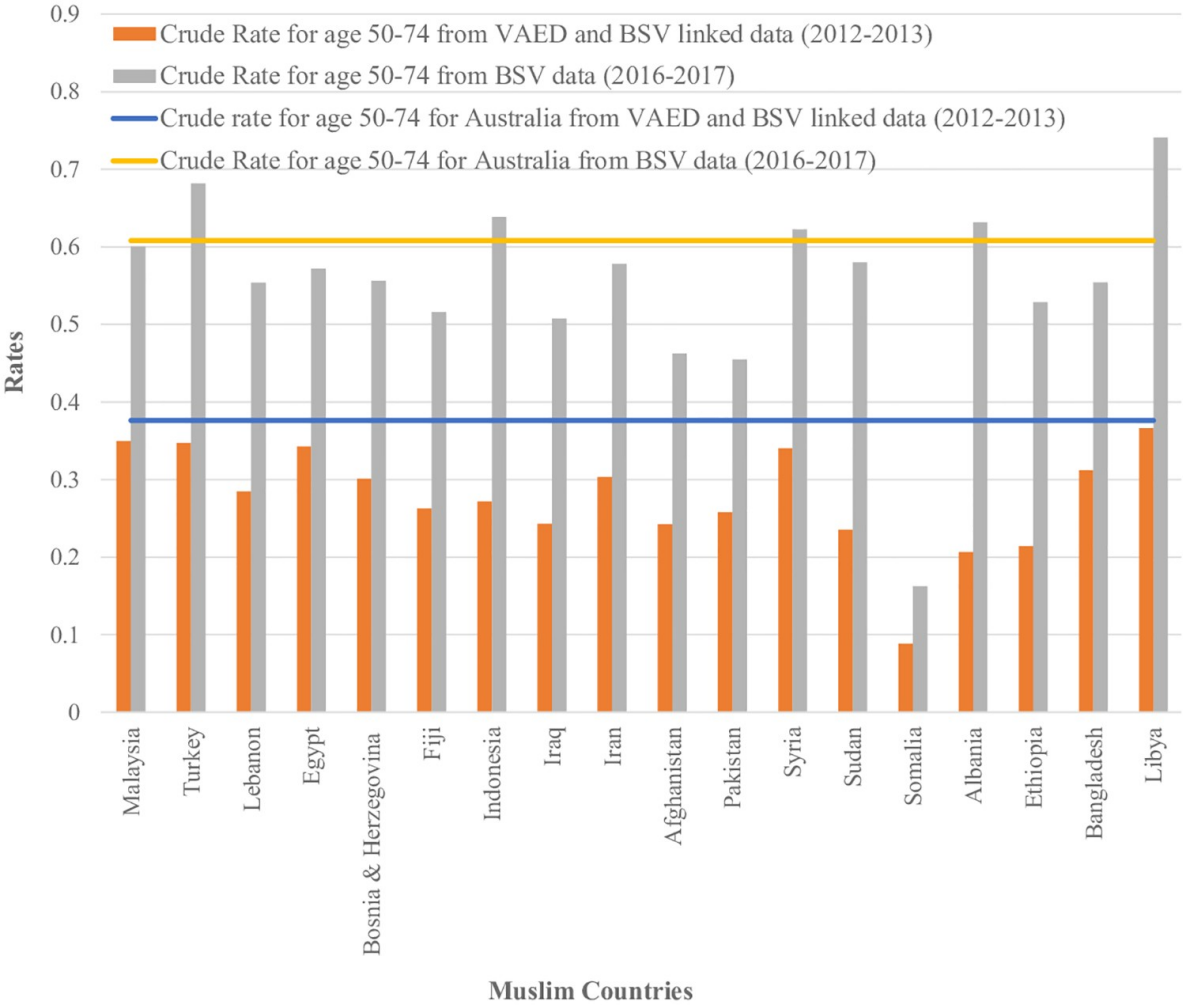
Comparative analysis of screening rate estimates for women aged 50–74 born in Muslim countries from both linked and unlinked datasets with their Australian counterpart (Countries with more than 100 women were reported only). Data Sources: Crude rate for age 50–74 from VAED and BSV linked data for women who were born in Muslim countries and Australia (Numerator from Breast Screen Victoria (BSV) registry linked with Victorian Admitted Episodes Dataset (VAED) and Denominator from VAED). Crude rate for age 50–74 from BSV data for women who were born in Muslim countries and Australia (Numerator from BSV and Denominator from ABS census estimates, obtained via the Victorian Department of Health and Human Services).

**Fig 5:**
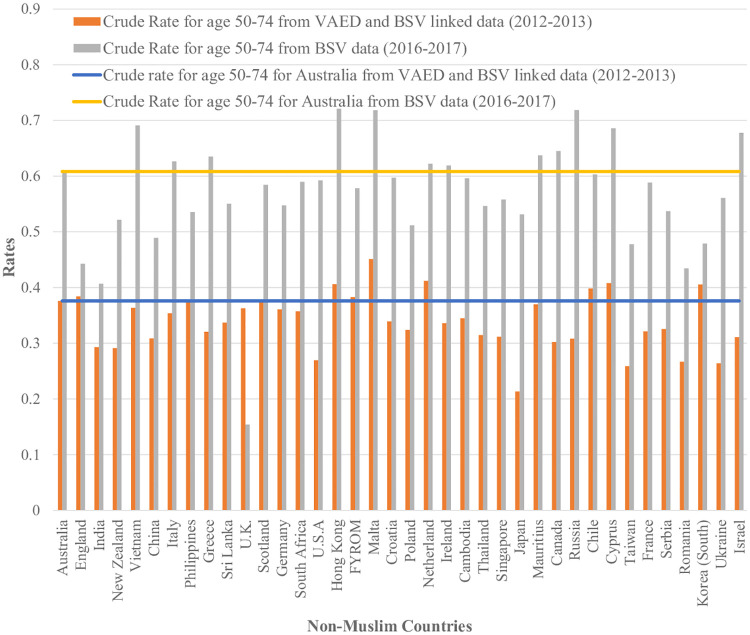
Comparative analysis of screening rate estimates for women aged 50–74 born in Non-Muslim countries from both linked and unlinked datasets with their Australian counterpart (Countries with more than 100 women were reported only). Data Sources: Crude rate for age 50–74 from VAED and BSV linked data for women who were born in Non- Muslim countries and Australia (Numerator from Breast Screen Victoria (BSV) registry linked with Victorian Admitted Episodes Dataset (VAED) and Denominator from VAED). Crude rate for age 50–74 from BSV data for women who were born in Non-Muslim countries and Australia (Numerator from BSV and Denominator from ABS census estimates, obtained via the Victorian Department of Health and Human Service).

In our study validation using BSV data for 2016–2017, we also found that estimated screening rates were generally lower for women who were born in Muslim countries (Figs 4 and 5).

## Discussion

Our analysis rests on the linkage quality of the two data sets whereby records in the VAED that were successfully matched to the BSV data are women correctly identified as having screened. Women in the VAED with no match in the BSV data set were assumed to have no screening record. Here we studied the breast screening participation status of women who were born in Muslim countries and residing in Victoria. We identified that the apparent screening rates (ASR) obtained through data linkage in this cohort were lower for women who were born in Muslim countries than for any other population group. In particular, women who were born in Muslim countries have lower ASR than women who were born in Australia across all age groups (Fig 1).

Prior studies have also found that women from culturally and linguistically diverse groups or socioeconomically disadvantaged areas have lower participation rate in breast screening [19]. Several studies have observed lower participation rates among Muslim women. Factors found in these studies that may have contributed to lower participation rates among Muslim women include positive religious coping (for e.g., use of religious support such as prayer to overcome stresses), self-efficacy [10], perceived religious discrimination in healthcare [12], cultural beliefs, limited knowledge and education [10,20]. Our study suggests that there is considerable diversity in ASR among women who were born in different Muslim countries.

Our study has several limitations. One potential limitation of our methodology is the likely under linkage between the Victorian Admitted Episodes Dataset (VAED) and Breast Screen Victoria (BSV) registry data. The VAED dataset did not contain names and addresses of the participants to match with BSV which could possibly result in under-matching between the two datasets. This is particularly a risk where women move residence over time (postcode will be different). When a screening record is not identified as a match to a woman in the VAED dataset when there is a record for that woman, then in our analysis we will mistakenly classify the woman as not screened. However, since the comparison is made across population groups, this under linkage will not affect the conclusions of this study about relative participation as long as an under matching is equally likely across the groups. Our cohort also consisted of women who had ever been hospitalized. Thus, the cohort might be biased towards women with worse health so that they have different screening behaviour compared to other women in the population.

However, our validation analysis using BSV participation data estimating screening rates in 2016–2017 is consistent with our overall finding of generally lower participation amongst women from Muslim countries with the exception of women born in Turkey, Indonesia, Syria, Albania and Libya (Fig 4). Data are not directly comparable due to the different time periods and methods but the magnitude of the difference in screening rates between Muslim women and non-Muslim women was similar using each method. The lower apparent screening rates for all women in the linked data is likely to be due to the estimation of screening across the age range of 45 to 84 rather than just for the target age range in 2012–13 which was 50–69 years (changed to 50–74 in July 2013) as well as to under linkage, given that the screening rate for women in Victoria in 2012–13 for aged 50–69 was 54.9% [21]. The availability of BSV data, with 99% of women in the BSV register reporting a country of birth, and their use in this project suggests that it would be valuable to utilise census estimates, at least periodically, for estimating screening participation rates by country of birth.

Another limitation of our approach is that Muslim women were identified based on their country of birth. However, some countries may have a significant presence of women from other religions. Our findings about the screening behaviour of Muslim women may not be generalized for all women from these countries. Although lower screening rates were observed among women born in Muslim countries, specific reasons for such lower participation were hard to find from the linked data. Qualitative analysis based on survey data can provide deeper insights on the reasons behind lower screening participation rates.

Women born in different Muslim countries have significantly different screening participation rates. For example, among all the Muslim countries Somalia has the lowest ASR. Lack of awareness about breast screening could be a potential reason for lower participation. Another potential reason will be lack of knowledge about the local health system. For instance, women born in Syria, Egypt, Turkey and Malaysia have relatively higher ASR compared to women born in other Muslim countries. It could be the case that women born in these countries have better knowledge about the Australian health care system and the availability of screening services than women born in other Muslim countries. It is also likely that the differences in cultural values over religious affiliation may play a role in the differences in screening participation [22]. Instead of visiting a general practitioner, for breast screening women have to make an appointment and travel to a different facility. Women born in Muslim countries may depend on other family members to access these services as they do not usually drive or travel alone. Lack of access to transport services for mammogram may lower their participation rate [23].

Previous studies from Australia, US [10,12,24] and UK [25] have found lower rates of breast screening amongst Muslim women supporting our results. These studies have investigated the role of religious and cultural differences in lower screening participation. Inadequacy of knowledge, awareness and doctor’s recommendation, fear, embarrassment and religious and cultural beliefs were responsible for Muslim women’s perception about breast screening [26–30]. By conducting a study over Muslim women in the USA, Padela and colleagues [12] found that women who were optimistic about religious coping and who fear about perceived religious discrimination in healthcare were responsible for lower rates of breast screening.

We also investigated if the lower participation rate of women who were born in Muslim countries still holds when factors such as socio-economic status, marital status and age are considered. After controlling for these factors, we found that women who were born in Muslim countries still have lower likelihood to participate in breast screening compared to women born in Australia and Non-Muslim countries. Furthermore, we found women from medium socio-economic categories were more likely to participate in breast screening compared to women from other socio-economic categories. However, studies investigating the association between area based socioeconomic measures and breast screening have found no consistent pattern [31]. In Australia, it is possible that more affluent women chose to use private providers for mammographic screening rather than utilise the free BreastScreen program.

This study has implications for breast screening programs as it highlights the need for culturally sensitive approaches to increase breast screening participation among women who were born in Muslim countries. The Victorian cancer plan 2016–2020 is already focusing on increasing equity in screening participation by implementing more accessible services for culturally and linguistically diverse communities [19]. The Ophelia (optimise health literacy and access) BreastScreen study found that emotional barriers are a significant factor in breast screening participation [32]. According to this study, compared to the women who speak English and Italian, women who speak Arabic reported more emotional barriers such as, lack of perception to be understood and lack of support from healthcare providers and understanding health information. Screening programs can provide support to practitioners to help overcome such barriers, as well as design culturally sensitive campaign strategies to raise awareness among women who were born in Muslim countries. BreastScreen Victoria aims to ensure equitable participation by all eligible Victorians. BreastScreen Australia’s National Accreditation Standards protocols for access and participation require services to identify under-screened groups and develop a recruitment plan to reduce barriers and increase awareness and participation.

This study provides valuable insights to understanding breast screening participation status among women who were born in Muslim countries and residing in Victoria and supports the ongoing need for screening programs to engage with the ethnically diverse population group, particularly the Muslim immigrant community.

## Supporting information

S1 Appendix List of Muslim countries. (DOCX)
