# Supplementary material for: Understanding the participation of breast screening among women born in predominantly Muslim countries living in Victoria, Australia from record-linkage data
Source: PLoS One. 2020 Aug 7;15(8):e0237341. doi: 10.1371/journal.pone.0237341 (PMC7413407; doi:10.1371/journal.pone.0237341)
Supplement: S1 Appendix — (DOCX) [file pone.0237341.s001.docx]

**S1 Appendix A. List of Muslim Countries-**

- Malaysia
- Turkey
- Lebanon
- Indonesia
- Iraq
- Fiji
- Egypt
- Bosnia & Herzegovina
- Sudan
- Afghanistan
- Pakistan
- Iran
- Ethiopia
- Somalia
- Bangladesh
- Syria
- Saudi Arabia
- Albania
- Nigeria
- Kuwait
- Libya
- Jordan
- UAE
- Morocco
- Ghana
- Uganda
- Brunei D
- Tanzania
- Uzbekistan
- Sierra Leon
- Kazakhstan
- Mauritania
- Bahrain
- Maldives
- Algeria
- Kosovo
- Western S
- Oman
- Azerbaijan
- Yemen
- Guyana
- Mozambique
- Djibouti
- Tunisia
- Qatar
- Gaza
- Kyrgyzstan
- Senegal
- Turkmenistan
- Togo
- Guinea
- Cameroon
- Tajikistan
- Niger
- Mali
- Comoros
- Chad
- Suriname
- Gambia
- Gabon
